# Preclinical efficacy of stem cell therapy for skin flap: a systematic review and meta-analysis

**DOI:** 10.1186/s13287-020-02103-w

**Published:** 2021-01-07

**Authors:** Yuan Li, Qi-lin Jiang, Leanne Van der Merwe, Dong-hao Lou, Cai Lin

**Affiliations:** 1 Department of Burn, The First Affiliated Hospital of Wenzhou Medical University, Nan Bai Xiang, Wenzhou, Zhejiang, 325000 People’s Republic of China; 2 School of International Studies, Wenzhou Medical University, Wenzhou, Zhejiang, 325000 People’s Republic of China

**Keywords:** Meta-analysis, Preclinical evidence, Skin flaps, Stem cell

## Abstract

**Background:**

A skin flap is one of the most critical surgical techniques for the restoration of cutaneous defects. However, the distal necrosis of the skin flap severely restricts the clinical application of flap surgery. As there is no consensus on the treatment methods to prevent distal necrosis of skin flaps, more effective and feasible interventions to prevent skin flaps from necrosis are urgently needed. Stem therapy as a potential method to improve the survival rate of skin flaps is receiving increasing attention.

**Methods:**

This review followed the recommendations from the Preferred Reporting Items for Systematic Reviews and Meta-Analysis (PRISMA) statements. Twenty studies with 500 animals were included by searching Web of Science, EMBASE, PubMed, and Cochrane Library databases, up until October 8, 2020. Moreover, the references of the included articles were searched manually to obtain other studies. All analyses were conducted using Review Manager V.5.3 software.

**Results:**

Meta-analysis of all 20 studies demonstrated stem cell treatment has significant effects on reducing necrosis of skin flap compared with the control group (SMD: 3.20, 95% CI 2.47 to 3.93). Besides, subgroup analysis showed differences in the efficacy of stem cells in improving the survival rate of skin flaps in areas of skin flap, cell type, transplant types, and method of administration of stem cells. The meta-analysis also showed that stem cell treatment had a significant effect on increasing blood vessel density (SMD: 2.96, 95% CI 2.21 to 3.72) and increasing the expression of vascular endothelial growth factor (VEGF, SMD: 4.34, 95% CI 2.48 to 6.1).

**Conclusions:**

The preclinical evidence of our systematic review indicate that stem cell-based therapy is effective for promoting early angiogenesis by up regulating VEGF and ultimately improving the survival rate of skin flap. In summary, small area skin flap, the administration method of intra-arterial injection, ASCs and MSCs, and xenogenic stem cells from humans showed more effective for the survival of animal skin flaps. In general, stem cell-based therapy may be a promising method to prevent skin flap necrosis.

## Introduction

A skin flap is one of the most critical surgical techniques for the restoration of cutaneous defects caused by trauma, tumor excision, lower limb vascular ulcer, or diabetes mellitus [1–3]. However, for skin flaps, especially for the treatment of large areas, distal necrosis is one of the most common postoperative complications [4]. This complication makes the ratio of length to width of the flap to be 1.5–2, which severely restricts the clinical application of flap surgery [5]. Clinical experience has shown that once the skin flap becomes necrotic, it will not only lead to the increase of possible secondary surgery and treatment costs, but also more pain and suffering [6]. Further, the main mechanisms of flap necrosis are insufficient blood perfusion, venous return disorder, and ischemia-reperfusion injury. If the degree of ischemia exceeds the tolerance threshold of the tissue without intervention in a short period of time, the ischemic part of the tissue will undergo irreversible necrosis [7]. To prevent necrosis of the skin flap, it is the key to improve local neovascularization and increase the blood supply to ischemic tissues. Therefore, appropriate exogenous intervention is necessary to accelerate early angiogenesis to prevent postoperative necrosis of the flap [8]. To make more flaps survive successfully, various strategies for preventing skin flap necrosis have recently been developed, including reduction of oxidative stress [9], inhibition of apoptosis [10], and vasodilators [11]. However, due to the bad effects of all the above mentioned treatment methods, there is no consensus on the treatment methods to prevent distal necrosis of skin flaps. Therefore, more effective and feasible interventions to prevent skin flaps from necrotizing are urgently needed.

Angiogenesis in skin flaps is an intricate process involving the coordination of various cells and cytokines [12]. Stem cells have the unique ability of self-renewal and differentiation into different cells, which provides a new possibility for regenerative medicine [13, 14]. Furthermore, many studies have revealed that stem cell therapy has a significant effect in protecting the heart, brain, and kidneys from ischemic damage and improving prognosis [15–17]. With this in mind, cell therapy as a potential method to improve the survival rate of skin flaps is receiving increasing attention [8, 18, 19]. However, even many animal experiments focus on this topic; unfortunately, there is almost no research on its clinical efficacy, let alone become the gold standard for clinical treatment of skin flaps. What is more, animal experiments, a bridge between the bench and the bedside, help assess the efficacy of stem cells and clarify further mechanisms [20]. Even many studies have hitherto explored the role of stem cells for skin flaps in animals, their efficacy and mechanisms have not been systematically summarized. To ensure that this promising and evidence-based stem cell therapy may develop into future clinical practice for patients who need skin flap surgery, we have conducted a systematic review of those animal studies to investigate the efficacy of stem cells for skin flaps.

## Methods

This study followed the recommendations from the Preferred Reporting Items for Systematic Reviews and Meta-Analysis (PRISMA) statements [21]. The PRISMA 2009 checklist was shown in Supplementary Table 1. Our protocol was published through PROSPERO (CRD42020213388) and also can be found online at https://www.crd.york.ac.uk/prospero/display_record.php? RecordID=213388z.

### Search strategy

Multiple databases (including PubMed, Web of Science, EMBASE, and Cochrane libraries) have been searched for related studies on improving the survival rate of skin flaps by stem cell therapy, up until October 8, 2020. We selected the following terms: (1) “mesenchymal stem cell” OR “progenitor cells” OR “mononuclear cells” OR “stem cell(s)” OR “mesenchymal stem cell” OR “Mesenchymal Stromal Cells” OR “adipose-derived stem cells” AND (2) “skin flap” OR “skin flaps”. All articles are limited to preclinical studies and published in English. Moreover, the references of the included articles were searched manually to obtain other studies.

### Eligibility criteria

The selection criteria for this study were prespecified as follows: (1) published as an original research article, (2) experimental models of skin flaps, (3) treatment groups treated with stem cells, (4) control group only received the liquid without therapeutic effect or no treatment, and (5) the primary outcome was the survival rate of skin flaps. The second outcome measures were blood vessel density and expression of vascular endothelial growth factor (VEGF). The exclusion criteria of studies were prespecified as follows: (1) animal models unrelated to skin flaps, (2) the application of stem cell in combination with other treatment methods in the treatment group, (3) no control group, and (4) clinical trial, review article, and duplicate publication.

### Data extraction

The studies that did not meet the inclusion criteria were excluded after screening all the articles searched. Two authors read the full text independently and extracted the relevant data. The differences raised during this period were handled and resolved by a third author. The following details were recorded: (1) the first author, year, and country of studies; (2) the species and number of animals, and the area of the flap; (3) origin, type, and quantity of stem cells; (4) intervention measures of treatment group and control group; and (5) primary and secondary outcome measurements collected. When some data was presented only in the form of figures, we tried to contact the author for more detailed data. If we did not get the corresponding reply from the author, we used the digital ruler software to measure the pictures to obtain the data.

### Assessment of the risk of bias

The quality assessment was carried out by two research experts independently, and the possible differences were comprehensively evaluated according to the opinions of the third expert. The risk of bias in our animal studies was assessed by a minor modified 10-item scale. The following domains were assessed: (a) sequence generation, (b) baseline characteristics, (c) allocation concealment, (d) random housing, (e) blinding of investigators, (f) random outcome assessment, (g) blinding of outcome assessor, (h) complete outcome data, (i) selective outcome reporting, and (j) other sources of bias.

### Statistical analysis

All analyses were conducted using the Review Manager V.5.3 software. Outcomes were continuous data and presented as standardized mean difference (SMD) with 95% confidence interval when the scales of data are inconsistent. The results of the meta-analysis are presented with forest diagram. Heterogeneity and choice of effects models were probed with the Cochrane Q-statistic test and the *I*^2^-statistic test. When *I*^2^ > 50%, indicating that the included studies have significant heterogeneity, and a random-effect meta-analysis model is used. Instead, a fixed-effect model was adopted. Explore the source of the heterogeneity when inter-study heterogeneity was obvious, and sensitivity analysis or subgroup analysis was conducted if necessary. Funnel plots were drawn to intuitively investigate publication bias when there were no less than ten studies that reported the same outcome measurement.

## Results

### Study selection

**Fig. 1:**
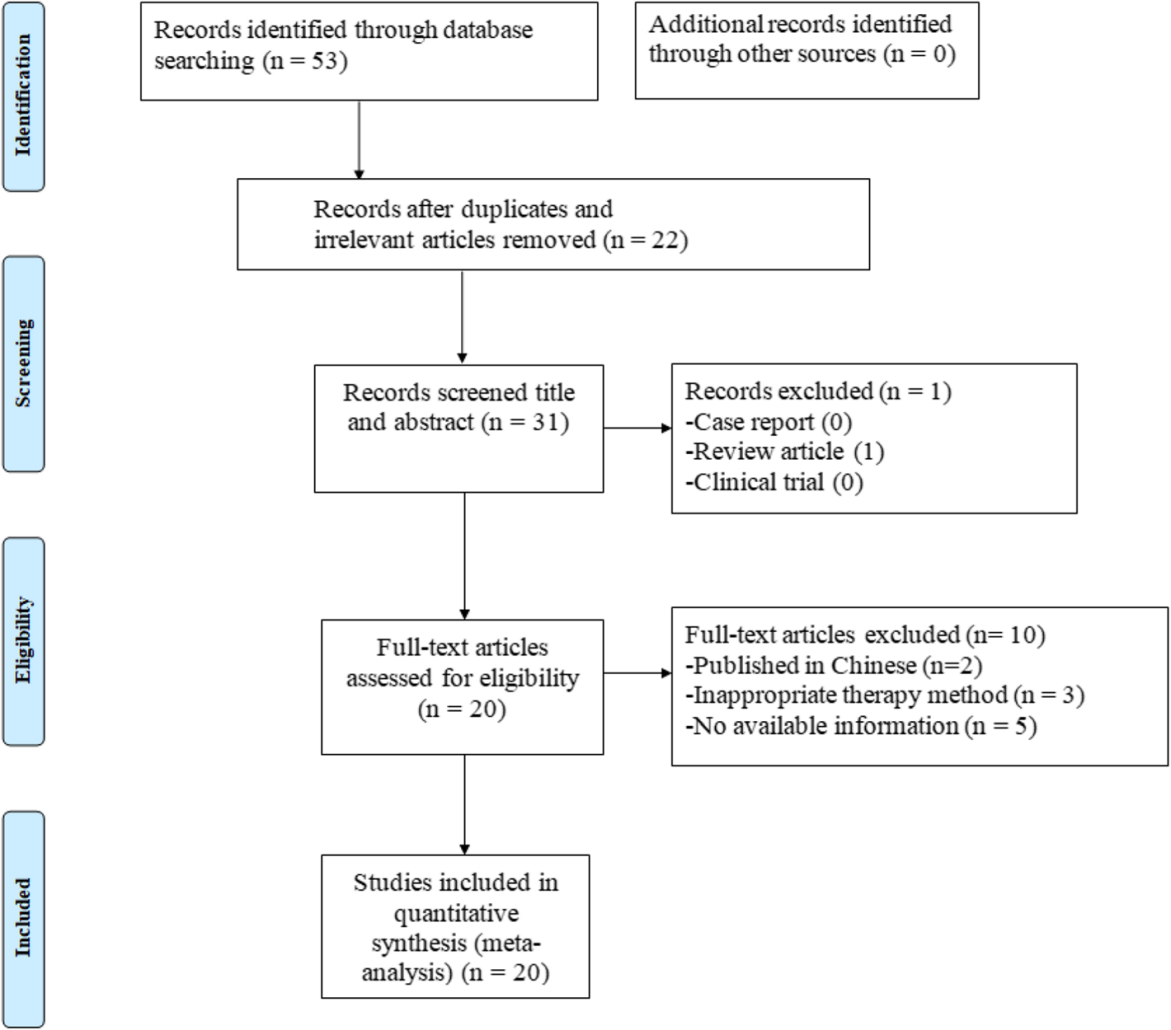
Flow diagram for the selection of studies, with article search strategy results

Our last search was conducted on October 1, 2020 (Fig. 1). A total of 53 potential hints were obtained in the initial search through the database. After excluding 22 irrelevant or reduplicated studies, the remaining 31 studies were further screened. After a screening of the title and abstract of the remaining studies and a careful reading of the full text, 11 studies were excluded for the following reasons: (1) review article [22], (2) published in Chinese [23, 24], (3) inappropriate therapy method [25–27], and (4) no available information [28–32]. Finally, twenty studies were included, and a meta-analysis of stem cells for skin flaps was conducted [8, 18, 19, 28, 33–48].

### Characteristics of included studies

A total of 20 studies and 24 treatment groups were included, and further data extraction was performed according to the classification listed in Table 1. These studies were published between 2010 and 2020. In this meta-analysis, a total of nine studies were completed in China [18, 19, 33, 34, 40, 41, 43–45], four in Iran [8, 46–48], four in Korea [28, 38, 39, 42], and the remaining three in Germany [37], Brazil [36], and the USA [36]. For animal selection, 60% of the experiments used mice as models [8, 28, 33, 34, 36, 37, 41, 42, 45–48], and 40% of the studies used rats [18, 19, 35, 38–40, 43, 44]. Most studies used random skin flap, and only three studies used axial skin flap [37, 40, 45]. The area of the skin flaps varies from 1.25 to 40 cm^2^ in the selected studies. Cell types were also compared, such that 11 of the included studies used MSCs, nine used ASCs, and one used MNCs [33]. It is worth mentioning that one study used both adipose-derived stem cells and mononuclear cells [33]. In terms of transplant type, 12 studies used allogenic cells [8, 33–37, 39, 42, 45–48], and eight studies used xenogenic cells [18, 19, 28, 38, 40, 41, 43, 44]. The number of cells given ranged from 10^3^ to 6 × 10^9^. The mode of administration varied by study, such that 75% of 24 treatment arms used subcutaneous injection, 12.5% intra-arterial injection, and 12.5% intravenous injection. The intervention measures in the control group were mainly phosphate-buffered saline (PBS) [36, 38, 44, 45], medium [19, 33, 34, 37, 40, 42, 43], saline [8, 46, 47], and no treatment [18, 28, 35, 39, 41, 48]. For outcome measures, survival rate of the flap was used in all studies, blood vessel density in ten studies [18, 19, 28, 34, 37, 39, 40, 43, 44, 48], and expression of vascular endothelial growth factor (VEGF) in six studies [8, 33, 34, 40, 43, 44].

**Table 1:** Characteristics of the included studies

Study (year)	Country	Animal (number)	Type of skin flap	Skin flap (cm^2^)	Cell type	Tissue of origin	Transplant type	Cell number	Method of administration	Placebo	Outcome index
Chehelcheraghi et al., 2020 [48]	Iran	Wistar rats (10)	Random	24	MSCs	Bone marrow	Allogenic	6 × 10^9^	Subcutaneous injection	None	1. Survival rate of flap 2. Blood vessel density
Chehelcheraghi et al., 2019 [8]	Iran	Wistar rats (20)	Random	24	MSCs	Bone marrow	Allogenic	6 × 10^9^	Subcutaneous injection	Saline	1. Survival rate of flap 2. VEGF
Chehelcheraghi et al., 2016 [47]	Iran	Wistar rats (20)	Random	24	MSCs	Bone marrow	Allogenic	1 × 10^9^	Subcutaneous injection	Saline	1. Survival rate of flap
Chehelcheraghi et al., 2015 [46]	Iran	Wistar rats (20)	Random	24	MSCs	Bone marrow	Allogenic	1 × 10^9^	Subcutaneous injection	Saline	1. Survival rate of flap
Ding et al. #1, 2020 [45]	China	Wistar rats (12)	Axial	25	MSCs	Bone marrow	Allogenic	1 × 10^6^	Subcutaneous injection	PBS	1. Survival rate of flap
Ding et al. #2, 2020 [45]	China	Wistar rats (12)	Axial	25	MSCs	Bone marrow	Allogenic	5 × 10^6^	Subcutaneous injection	PBS	1. Survival rate of flap
Feng et al. #1, 2020 [44]	China	BALB/C mice (20)	Random	9	ASCs	Adipose	Xenogenic	1 × 10^3^	Intra-arterial injection	PBS	1. Survival rate of flap 2. Blood vessel density 3. VEGF
Feng et al. #2, 2020 [44]	China	BALB/C mice (20)	Random	9	ASCs	Adipose	Xenogenic	1 × 10^4^	Intra-arterial injection	PBS	1. Survival rate of flap 2. Blood vessel density 3. VEGF
Feng et al. #3, 2020 [44]	China	BALB/C mice (20)	Random	9	ASCs	Adipose	Xenogenic	1 × 10^5^	Intra-arterial injection	PBS	1. Survival rate of flap 2. Blood vessel density 3. VEGF
Gao et al., 2011 [43]	China	BALB/c mice (30)	Random	3	ASCs	Adipose	Xenogenic	1 × 10^7^	Subcutaneous injection	Medium	1. Survival rate of flap 2. Blood vessel density 3. VEGF
Han et al., 2015 [42]	Korea	SD rats (14)	Random	24	ASCs	Adipose	Allogenic	5 × 10^5^	Subcutaneous injection	Medium	1. Survival rate of flap
Leng et al., 2017 [41]	China	Wistar rats (48)	Random	18	MSCs	Umbilical cord	Xenogenic	4 × 10^5^	Subcutaneous injection	None	1. Survival rate of flap
Leng et al., 2012 [40]	China	BALB/c mice (20)	Axial	18	MSCs	Umbilical cord	Xenogenic	4 × 10^5^	Subcutaneous injection	Medium	1. Survival rate of flap 2. Blood vessel density 3. VEGF
Moon et al., 2018 [39]	Korea	ICR mice (16)	Random	4.5	MSCs	Bone marrow	Allogenic	2 × 10^6^	Subcutaneous injection	None	1. Survival rate of flap 2. Blood vessel density
Pak et al., 2020 [28]	Korea	SD rats (12)	Random	27	ASCs	Adipose	Xenogenic	5 × 10^6^	Subcutaneous injection	None	1. Survival rate of flap 2. Blood vessel density
Park et al., 2017 [38]	Korea	BALB/c mice (16)	Random	8	ASCs	Adipose	Xenogenic	1.5 × 10^6^	Subcutaneous injection	PBS	1. Survival rate of flap
Pu et al., 2017 [19]	China	C57BL/6 J mice (12)	Random	4	ASCs	Adipose	Xenogenic	1 × 10^6^	Subcutaneous injection	Medium	1. Survival rate of flap 2. Blood vessel density
Reichenberger et al., 2012 [37]	Germany	Lewis rats (16)	Axial	60	ASCs	Adipose	Allogenic	5 × 10^6^	Intravenous injection	Medium	1. Survival rate of flap 2. Blood vessel density
Suartz et al., 2014 [36]	Brazil	Wistar rats (30)	Random	40	ASCs	Adipose	Allogenic	5 × 10^6^	Intravenous injection	PBS	1. Survival rate of flap 2. Blood vessel density
Tang et al., 2016 [35]	USA	C57Bl6 mice (12)	Random	2	MSCs	Bone marrow	Allogenic	3 × 10^6^	Intravenous injection	None	1. Survival rate of flap 2. Blood vessel density
Wang et al., 2011 [34]	China	SD rats (20)	Random	16	MSCs	Bone marrow	Allogenic	4 × 10^6^	Subcutaneous injection	Medium	1. Survival rate of flap 2. Blood vessel density 3. VEGF
Yang et al. #1, 2010 [33]	China	Wistar rats (20)	Random	27	MNCs	Bone marrow	Allogenic	1 × 10^8^	Subcutaneous injection	Medium	1. Survival rate of flap 2. VEGF
Yang et al. #2, 2010 [33]	China	Wistar rats (20)	Random	27	ASCs	Adipose	Allogenic	4 × 10^6^	Subcutaneous injection	Medium	1. Survival rate of flap 2. VEGF
Zhou et al., 2019 [18]	China	BALB/C mice (40)	Random	1.25	MSCs	Bone marrow	Xenogenic	2.5 × 10^4^	Subcutaneous transplantation	None	1. Survival rate of flap 2. Blood vessel density

*ASCs* adipose-derived stem cells, *ICR* imprinting control region, *MNCs* mononuclear cells, *MSCs* mesenchymal stem cells, *NA* not available, *PBS* phosphate-buffered saline, *SD* Sprague-Dawley, *VEGF* vascular endothelial growth factor

### Quality of included studies

As evaluated by a modified 10-item scale, the 20 included studies were medium-quality animal experiments. The risk of biases for all the included studies is shown in Table 2. Few studies did not use random allocation [19, 28, 35, 45], and all studies reported baseline characteristics but did not allocation concealment. It should be noted that random housing is not mentioned in all studies. Under the domain for “blinding of investigators,” two studies were assessed low risk of bias [37, 44], while the rest of the studies were considered as unclear risk of bias. Seven studies described the use of random outcome for assessment [33, 34, 39–42, 44], and eight studies conducted a blind method to outcome assessor [18, 37, 39, 40, 42–44, 48]. In our meta-analysis, all studies are considered to report the outcome data completely and avoid outcome. Notably, there was uncertainty regarding the other sources of bias.

**Table 2:** Risk of bias of the included studies

Study	A	B	C	D	E	F	G	H	I	J	Toal
Chehelcheraghi et al., 2020 [48]	+	+	–	?	?	?	+	+	+	?	5
Chehelcheraghi et al., 2019 [8]	+	+	–	?	?	?	?	+	+	?	4
Chehelcheraghi et al., 2016 [47]	+	+	–	?	?	?	?	+	+	?	4
Chehelcheraghi et al., 2015 [46]	+	+	–	?	?	?	?	+	+	?	4
Ding et al., 2020 [45]	?	+	–	?	?	?	?	+	+	?	3
Feng et al., 2020 [44]	+	+	–	?	+	+	+	+	+	?	7
Gao et al., 2011 [43]	+	+	–	?	?	?	+	+	+	?	5
Han et al., 2015 [42]	+	+	–	?	?	+	+	+	+	?	6
Leng et al., 2017 [41]	+	+	–	?	?	+	?	+	+	?	5
Leng et al., 2012 [40]	+	+	–	?	?	+	+	+	+	?	6
Moon, 2018 [39]	+	+	–	?	?	+	+	+	+	?	6
Pak et al., 2020 [28]	?	+	–	?	?	?	?	+	+	?	3
Park et al., 2017 [38]	+	+	–	?	?	?	?	+	+	?	4
Pu et al., 2017 [19]	?	+	–	?	?	?	?	+	+	?	3
Reichenberger et al., 2012 [37]	+	+	–	?	+	?	+	+	+	?	5
Suartz et al., 2014 [36]	+	+	–	?	?	?	?	+	+	?	4
Tang et al., 2016 [35]	?	+	–	?	?	?	?	+	+	?	3
Wang et al., 2011 [34]	+	+	–	?	?	+	?	+	+	?	5
Yang et al., 2010 [33]	+	+	–	?	?	+	?	+	+	?	5
Zhou et al., 2019 [18]	+	+	–	?	?	?	+	+	+	?	5

Studies fulfilling the criteria of *A* sequence generation, *B* baseline characteristics, *C* allocation concealment, *D* random housing, *E* blinding of investigators, *F* random outcome assessment, G blinding of outcome assessor, *H* complete outcome data, *I* selective outcome reporting, and *J* other sources of bias

### Effect size

#### Primary outcome measures

##### Survival rate of flap

**Fig. 2:**
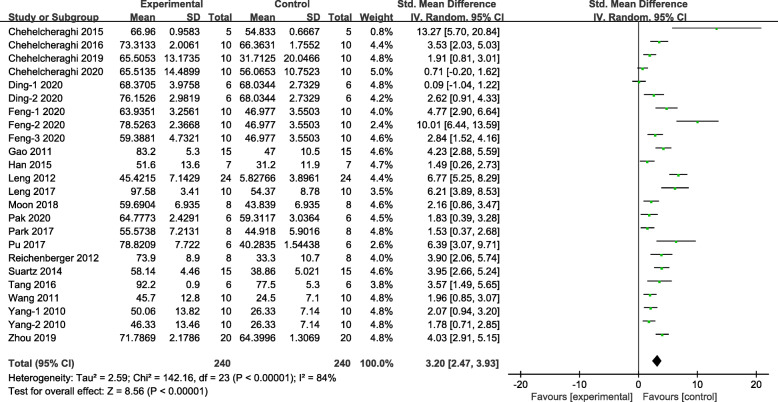
The forest plot: the effects of stem cell therapy for increasing survival rate of skin flaps compared with controls (*n* = 240 per group)

Survival rate of flap, the most obvious result data, is used as the primary outcome measure in this systematic review. Meta-analysis of all 20 studies demonstrated stem cell treatment has significant effects on reducing necrosis of skin flap compared with control group (*n* = 480, SMD 3.20, 95% CI 2.47 to 3.93, *P* < 0.00001; *I*^2^ = 84%, Fig. 2).

Since significant heterogeneity was found in this meta-analysis, we conducted further analysis of the source of heterogeneity, including sensitivity and subgroup analysis. However, the results of the sensitivity analysis showed that the heterogeneity did not decrease significantly after excluding individual studies in turn. Thus, subgroup analysis was performed by grouping studies according to the following classification: type of skin flap, area of skin flap, cell type, cell number, transplant types, and method of administration of stem cells used in treatment. The dose of stem cells in the intervention groups and the area of skin flaps varied widely in the included studies. Therefore, we divided the dose of stem cells into low (< 5 × 10^6^ cells) and high (≥ 5 × 10^6^ cells) in advance and divided the area of skin flaps into small (< 10 cm^2^) and large (≥ 10 cm^2^). The results of the subgroup analysis showed that there was no obvious heterogeneity between the subgroups in terms of flap types (Fig. S1 Supplementary materials), stem cell number (Fig. S2 Supplementary materials), and treatment measures of the control group (Fig. S3 Supplementary materials). Notably, although all types have been proven to be effective to skin flaps, adipose-derived stem cells (ASCs; SMD 3.39) and mesenchymal stem cells (MSCs; SMD 3.29) are considered to show a statistically larger effect size than mononuclear cells (MNCs; SMD 2.07; Fig. S4 Supplementary materials). In the subgroup analysis of skin flap area, both small area (< 10 cm^2^) and large area (≥ 10 cm^2^) demonstrated the effect of stem cell therapy on skin flaps, with the former being more effective (SMD 3.88 vs 2.79; Fig. S5 Supplementary materials). Interestingly, in terms of skin flap survival rate, the therapeutic effect of xenogeneic stem cells is significantly better than that of allogeneic transplantation types (SMD 4.51 vs 2.35; Fig. S6 Supplementary materials). By comparing the studies of different administration routes, we discovered that intravascular injection, including intra-arterial injection (SMD 5.45, 95% CI 2.25 to 8.64) and intravenous injection (SMD 3.86, 95% CI 2.92 to 4.80), has a more significant effect in preventing skin flap necrosis, compared to subcutaneous injection (SMD 2.77, 95% CI 1.96 to 3.58; Fig. S7 Supplementary materials). However, this result may be affected by other factors. For example, only a few studies [35–37, 44] have used the method of administration of intravascular injection.

#### Secondary outcome measures

##### Blood vessel density

**Fig. 3:**
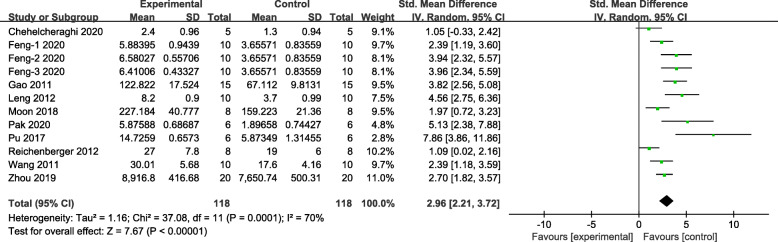
The forest plot: the effects of stem cell therapy for increasing blood vessel density of skin flaps compared with controls (*n* = 118 per group)

The meta-analysis of 12 groups demonstrated stem cell treatment has significant effects on increasing blood vessel density compared with control group (*n* = 236, SMD 2.96, 95% CI 2.21 to 3.72, *P* < 0.00001; *I*^2^ = 70%, Fig. 3).

##### VEGF

**Fig. 4:**
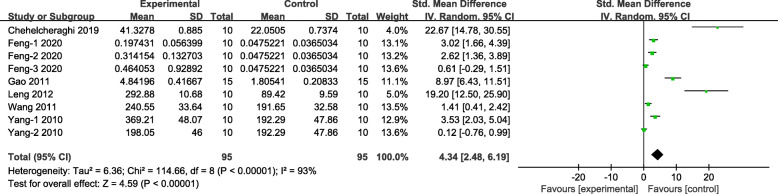
The forest plot: the effects of stem cell therapy for increasing the expression of VEGF in skin flaps compared with controls (*n* = 95 per group)

Meta-analysis of nine groups demonstrated stem cell-treated group was superior to the control group according to the increased the expression of VEGF (*n* = 190, SMD 4.34, 95% CI 2.48 to 6.19, *P* < 0.00001; *I*^2^ = 93%, Fig. 4).

#### Publication bias

**Fig. 5:**
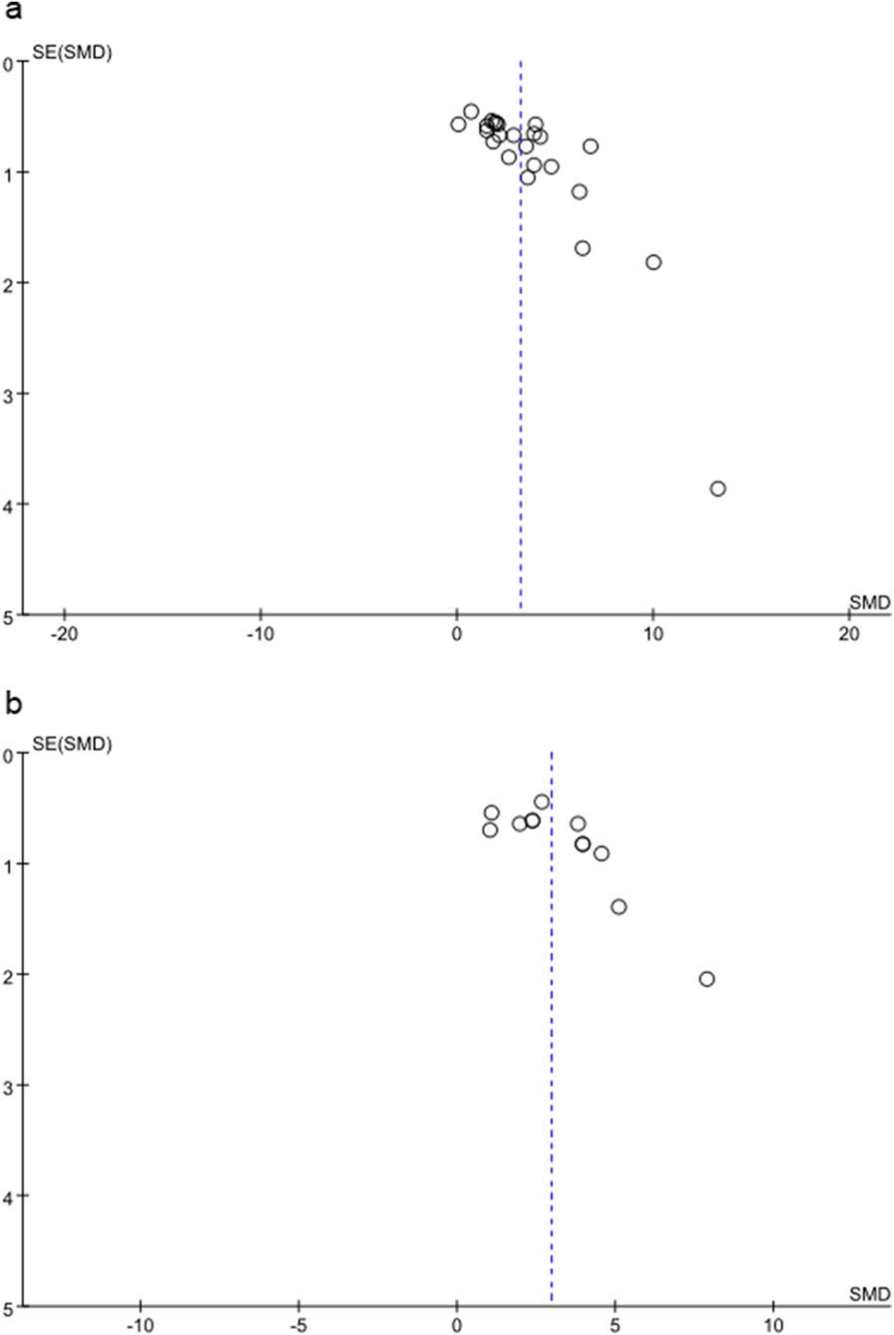
Funnel plot of included studies for **a** survival rate of flap and **b** blood vessel density

We evaluated publication bias of survival rate of skin flaps and blood vessel density by funnel plots. As shown in Fig. 5, the trend of the funnel plot of flap survival rate and blood vessel density is generally the same. The distribution of the funnel plots was slightly asymmetric, indicating that there may be potential publication bias.

## Discussion

As we all know, a free skin flap or pedicled skin flap transplantation is an indispensable key technology in numerous surgical fields to repair large tissue defects [49, 50]. Part of or all of skin flap necrosis is a common postoperative complication, and tissue ischemia caused by insufficient blood supply often leads to skin flap failure [51]. However, stem cells have the ability of self-renewal and differentiation into various cell lines, which provides the possibility of early angiogenesis of the skin flap [19, 52]. Therefore, the purpose of this review is to provide preclinical evidence for the effectiveness of stem cell therapy for skin flaps.

To our knowledge, this is the first preclinical systematic review and meta-analysis to estimate the efficacy and possible mechanism of stem cell therapy in promoting flap survival. The study is timely, considering that various stem cell therapeutic potentials are currently being tested in numerous preclinical trials, and clinical trials are almost not conducted. Our meta-analysis included 20 studies with 500 animals and analyzed three outcomes that were essential for the survival of the skin flap. Overall, the quality of the 20 studies included was moderate. In the present study, stem cell-based therapy effectively promotes early angiogenesis by upregulating VEGF and ultimately improving the survival rate of the skin flap. Angiogenesis in skin flaps is an intricate process involving the coordination of various cells and cytokines [12]. Rong et al. reported that human fetal skin-derived stem cell secretome promotes skin healing by activating the expression of specific genes related to angiogenesis, such as VEGF and placental growth factor (PLGF) [53]. Studies have indicated that exosomes secreted by mesenchymal stem cells may carry complex biological information, including mRNA and soluble proteins [54]. In addition, antler stem cells have been proven to stimulate fibrogenesis and angiogenesis to accelerate wound healing [55].

We found obvious heterogeneity in meta-analysis, so we further explored different research designs, including types of skin flap, area of skin flap, cell type, cell number, transplant types, and method of administration of stem cell used in treatment. However, the results may contribute to the future clinical transformation of stem cells to the bedside. Although different administration routes, cell types, skin flap areas, and species have significant effects in the prevention of skin flap necrosis, this study proves that stem cells are promising candidates for the prevention of skin flap necrosis.

At present, compared with MNCs (4.2% of all groups), MSCs (50%) and ASCs (45.8%) are the most frequently used. Our analysis pointed out that both adipocyte and mesenchymal stem cells showed comparable efficacy in terms of skin flap survival. MNCs are a group of cells composed of multiple progenitor cells/stem cells and other cell types. As it is abundant in the peripheral blood, it can be directly collected and applied to skin flap treatment. Similar to skin flaps, MNCs were also found to promote local capillary regeneration and vascular revascularization in infarcted limbs [56]. However, the effectiveness of monocytes for skin flap is significantly lower than that of the two cells mentioned above, and this result may also be affected by the limitation of only one study using such cells. Interestingly, Yang et al. [33] believed that the effectiveness of MNC transplantation in accelerating neovascularization of skin flaps may be related to basic fibroblast growth factor (bFGF) and VEGF secreted by MNCs. In clinical practice, MNCs have some characteristics, such as high safety without troublesome in vitro culture, as well as easy to isolate and obtain, which makes them have a great prospect in future research and application. However, there is growing evidence that the therapeutic mechanism of MSCs and ASCs is not only realized through paracrine cytokines and growth factors, but also can directly differentiate into vascular endothelial cells and skin components, thereby achieving vascular regeneration more effectively the goal of [57–60]. Although the most suitable type of stem cells for the clinical treatment of skin flaps cannot be directly concluded, preclinical evidence shows that MSCs and ASCs have better therapeutic effects with more trouble in their preparation process compared with MNCs. We also explored the effects of different transplant types of stem cells (Fig. S6). The subgroup analysis of transplant types showed that the efficacy of xenogeneic stem cells is significantly better than that of allogeneic stem cells applied to skin flaps (SMD 4.51 vs 2.35; Fig. S6 Supplementary materials). In recent years, the safety and effectiveness of human-derived stem cells used in clinical and preclinical studies have been confirmed [61]. This result seems to imply that HLA-matched stem cells from the donor-recipient may not be necessary to prevent skin flap necrosis.

The area of the skin flap also contributes to partial heterogeneity. In the subgroup analysis of skin flap area, both small area (< 10 cm^2^) and large area (≥ 10 cm^2^) demonstrated the effect of stem cell therapy on skin flaps, with the former being more effective (SMD 3.88 vs 2.79). That patients with more areas of skin flap usually require more nutrition for revascularization may explain this result. Besides, the larger the area of the skin flap is the greater the probability of infection, which will eventually lead to a larger area of necrosis. The most important thing is that the regenerative capacity of stem cells may have certain limitations, which makes the skin flap with a large area unable to survive more within the range of stem cell regeneration capacity. This result leads to a better effectiveness occurring on a smaller area of the skin flap. Consistent with our finding, a previous meta-analysis concluded that in the application of stem cells to treat burn wounds, smaller burn wounds are more likely to heal effectively [62]. Overall, the application of stem cells to skin flaps has shown considerable efficacy regardless of the area of the flap. It is foreseeable that stem cells can be considered as a promising treatment method for large-scale skin flaps in the clinic.

In addition, the delivery route was found to contribute to heterogeneity, accounting for 51.7%. In our included studies, 25% of the groups used vascular cell delivery (including intra-arterial injection [44] and intravenous injection [35–37]), and 75% of the groups used subcutaneous injection. In the method of administration subgroup analysis, both subcutaneous injection and intravascular injection showed the effect of stem cell therapy on skin flap, especially intra-arterial injection. Feng et al. [44] reported a significant reduction in the necrotic area of flaps after intra-arterial injection of ASCs; however, this result may be related to the axial type of skin flap used in this study. Considering that stem cell therapy aims to improve the survival rate of skin flap by promoting early angiogenesis, intra-arterial injection seems to be a reasonable and effective delivery route. We should also note that intra-arterial injection can cause vascular injury and other complications due to its invasiveness, which should be paid attention to in the future clinical operation. Although subcutaneous, intravenous, and intraarterial injections are commonly used in clinical practice, the best administration method is not yet known for sure due to the small number of studies using intravascular injection.

### Methodological considerations

The ultimate goal of preclinical research is to enrich our understanding of the causes and treatments of diseases and to lay the foundation for future clinical trials [63]. Significant research results depend on accurate preclinical research reports, while the defects in experimental design lead to low-quality reports that may exaggerate or weaken the effect sizes [64]. We put forward some views on preclinical studies to prevent unsuccessful translation. First, the studies we included did not use animals with comorbidities such as diabetes, hypertension, or vascular disease. In clinical practice, most people who need flap surgery are patients with these comorbidities. The selection of inappropriate animal models may lead to inconsistent results, limiting the development of preclinical research into clinical trials [65].

Second, enough attention should be paid to the quality of the methodology. In many fields, animal reports about the results of biomedical research are inadequate [66]. Effective research results depend on accurate preclinical research reports, while insufficient experimental design will lead to low-quality reports that may lead to exaggeration or neglect of the effect [64]. Since most of the published studies based on skin flaps treated with stem cells do not use blinding of investigators and outcome assessors, the overall quality of the research is not high. To improve the overall quality of future research, we recommend that the application of stem cells in skin flap related research should be double-blind. The quality of methodology was moderate in our included studies, so we suggest that ARRIVE guidelines should be referred to for further animal experiments and design [66].

### Limitations

Despite many advantages in our meta-analysis and systematic review, some potential limitations still existed and should to be considered when using the results. First of all, although our analysis does prove the significant efficacy of stem cell-based treatments on skin flaps, the heterogeneity between studies has to be mentioned. Therefore, we deal with substantial heterogeneity in the following aspects: (1) a random-effect model was used, (2) standardized mean difference was applied for all measurement outcomes, and (3) sensitivity analysis and subgroup analysis were performed to explore the sources of heterogeneity. Further subgroup analyses are needed to determine the most suitable source of stem cells, the appropriate method of administration, and the optimal type of transplantation. However, this analysis method will lead to a sharp reduction in the number of studies in each group, such as studies using intravenous injection and intra-arterial injection. More studies in the future can make the results of this study more stable and reliable. Secondly, most of the data is not easily obtained in digital form, but is extracted from graphs in published articles. The accuracy of the data will be affected by the distortion of the picture, but similarly, all groups will receive the same impact. Thirdly, we focused on the survival of skin flap as the primary outcome of our meta-analysis, with blood vessel density and expression of VEGF as the second outcome. Whether there are other essential mechanisms in the treatment of skin flap based on stem cells in addition to promoting the angiogenesis of skin flap still needs to be investigated. For example, cutaneous appendages in skin flaps should be further studied in future research. As cutaneous appendages including sebaceous glands and sweat glands play an important role in the skin function. Finally, possible publication bias was found in our study according to the qualitative results of funnel plots. Negative studies that were difficult to publish may contribute to publication bias, which may exaggerate the validity of the system evaluation.

## Conclusions

In conclusion, the preclinical evidence of our systematic review indicates that stem cell-based therapy is effective for promoting early angiogenesis by upregulating VEGF and ultimately improving the survival rate of the skin flap. We also found that preclinical data are significantly heterogeneous, and clinical application of stem cells is rarely explored, which makes the results need more exploration. However, the differences in this study are likely to contribute to the future clinical application of stem cells and have significant guidance for future translational and research projects. In summary, small area skin flap, the administration method of intra-arterial injection, ASCs and MSCs, and xenogenic stem cells from humans showed more effective for the survival of animal skin flaps. Besides, the quality of methodology and appropriate model selection should be paid more attention to in future research. In general, stem cell-based therapy may be a promising method to prevent skin flap necrosis.

## Supplementary Information

**Additional file 1.** PRISMA checklist for minimum set of items for reporting in systematic reviews and meta-analyses.

**Additional file 2: Figure S1.** Subgroup analyses of type of skin flap regarding stem cell therapy in animal model of skin flap for the primary outcome of survival rate of flap.

**Additional file 3: Figure S2.** Subgroup analyses of cell number regarding stem cell therapy in animal model of skin flap for the primary outcome of survival rate of flap.

**Additional file 4: Figure S3.** Subgroup analyses of treatment methods in the control group regarding stem cell therapy in animal model of skin flap for the primary outcome of survival rate of flap.

**Additional file 5: Figure S4.** Subgroup analyses of cell type regarding stem cell therapy in animal model of skin flap for the primary outcome of survival rate of flap.

**Additional file 6: Figure S5.** Subgroup analyses of area of skin flap regarding stem cell therapy in animal model of skin flap for the primary outcome of survival rate of flap.

**Additional file 7: Figure S6.** Subgroup analyses of transplant types regarding stem cell therapy in animal model of skin flap for the primary outcome of survival rate of flap.

**Additional file 8: Figure S7.** Subgroup analyses of method of administration of stem cells regarding stem cell therapy in animal model of skin flap for the primary outcome of survival rate of flap.

## Data Availability

The data supporting the conclusions of this article are all online.

## Abbreviations

ASCs: Adipose-derived stem cells
bFGF: Basic fibroblast growth factor
CI: Confidence interval
ICR: Imprinting control region
MNCs: Mononuclear cells
MSCs: Mesenchymal stem cells
NA: Not available
PBS: Phosphate-buffered saline
PLGF: Placental growth factor
SD: Sprague Dawley
SMD: Standard mean difference
VEGF: Vascular endothelial growth factor
