# Supplementary figures and images for: Preclinical efficacy of stem cell therapy for skin flap: a systematic review and meta-analysis
Source: Stem Cell Res Ther. 2021 Jan 7;12:28. doi: 10.1186/s13287-020-02103-w (PMC7791712; doi:10.1186/s13287-020-02103-w)

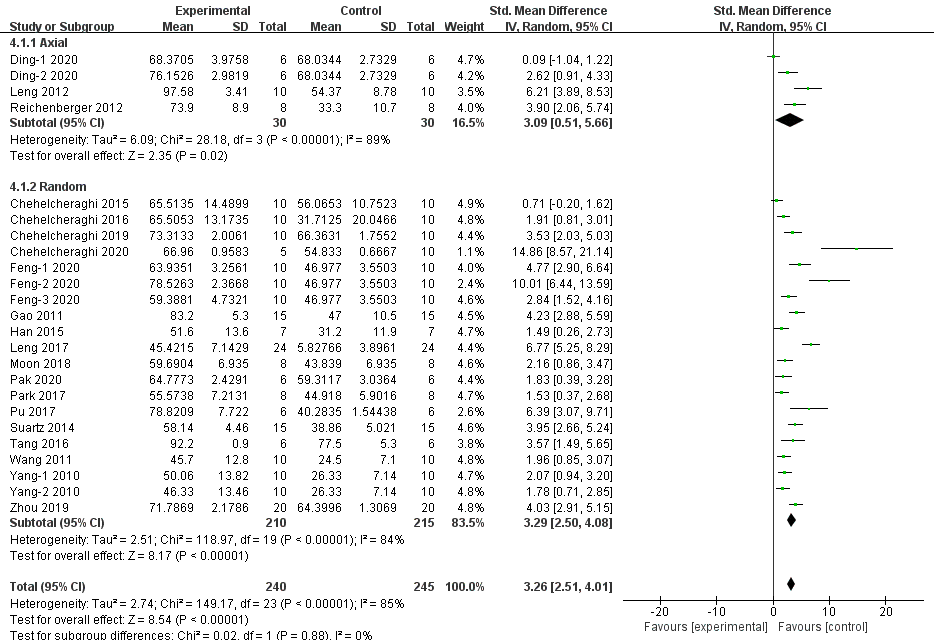

Supplement: Supplementary file 2 — Additional file 2: Figure S1. Subgroup analyses of type of skin flap regarding stem cell therapy in animal model of skin flap for the primary outcome of survival rate of flap. [file 13287_2020_2103_MOESM2_ESM.png]

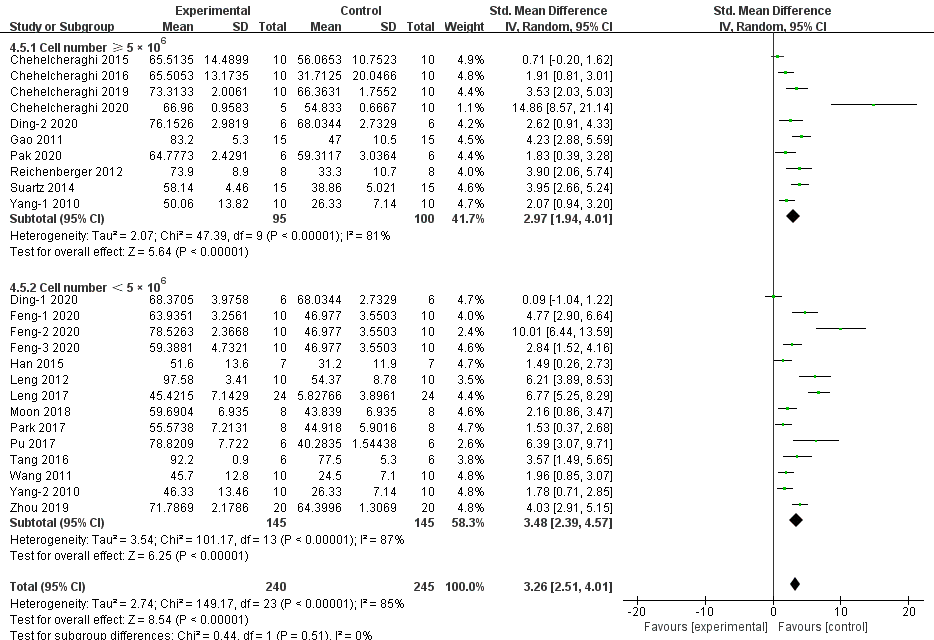

Supplement: Supplementary file 3 — Additional file 3: Figure S2. Subgroup analyses of cell number regarding stem cell therapy in animal model of skin flap for the primary outcome of survival rate of flap. [file 13287_2020_2103_MOESM3_ESM.png]

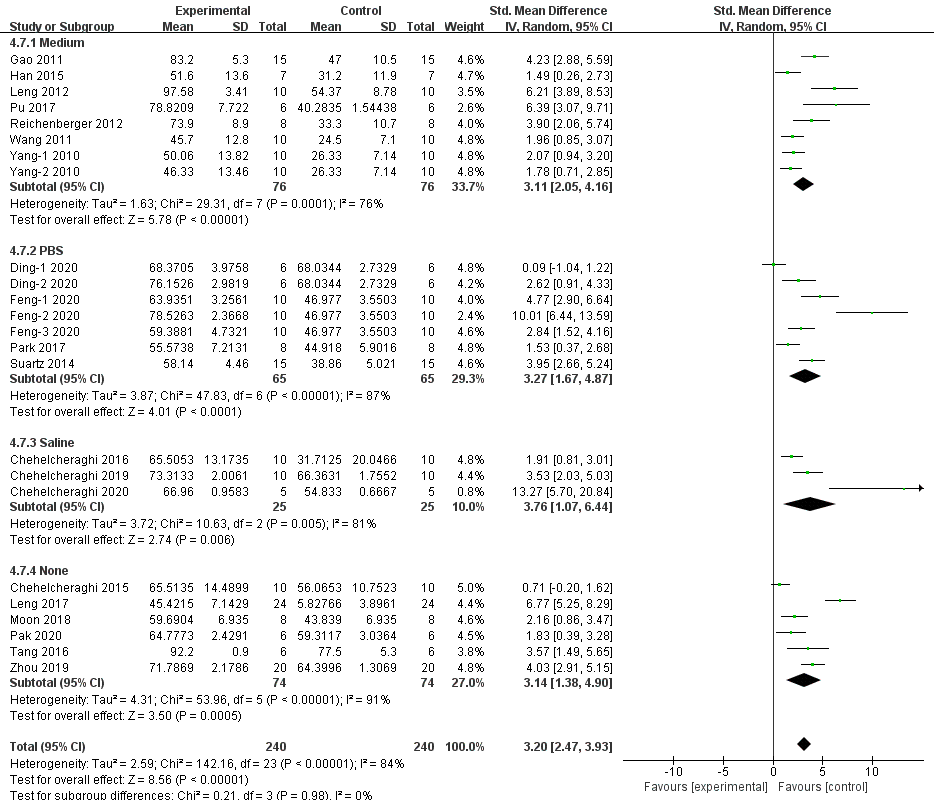

Supplement: Supplementary file 4 — Additional file 4: Figure S3. Subgroup analyses of treatment methods in the control group regarding stem cell therapy in animal model of skin flap for the primary outcome of survival rate of flap. [file 13287_2020_2103_MOESM4_ESM.png]

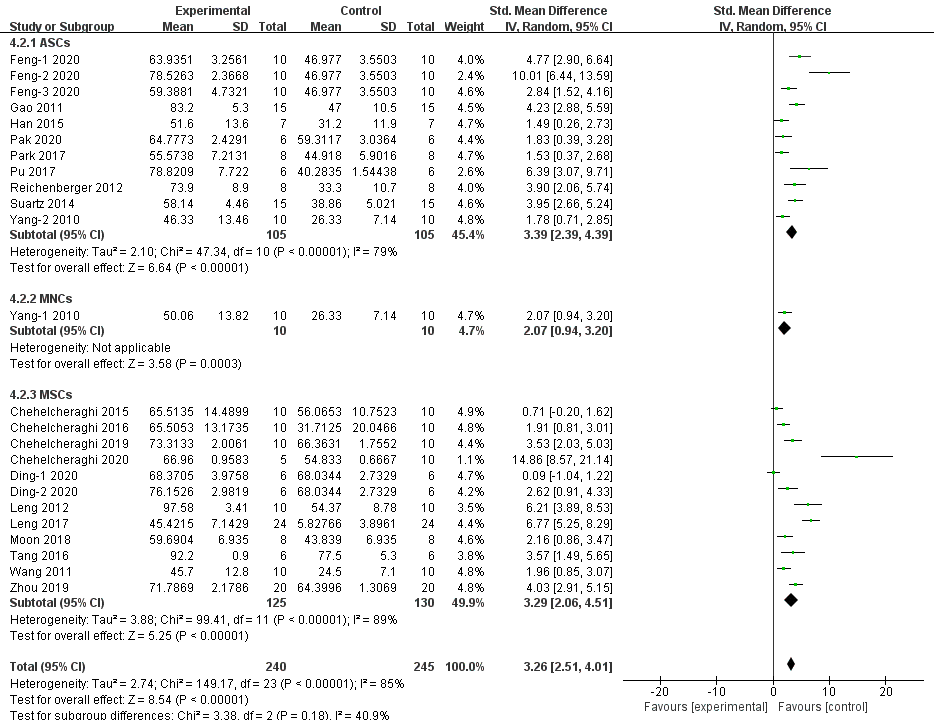

Supplement: Supplementary file 5 — Additional file 5: Figure S4. Subgroup analyses of cell type regarding stem cell therapy in animal model of skin flap for the primary outcome of survival rate of flap. [file 13287_2020_2103_MOESM5_ESM.png]

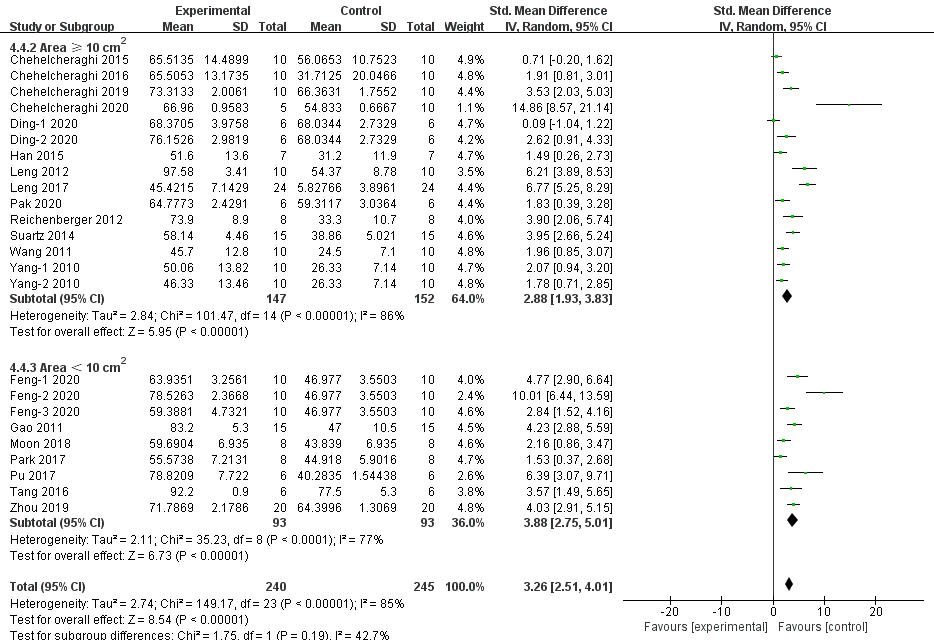

Supplement: Supplementary file 6 — Additional file 6: Figure S5. Subgroup analyses of area of skin flap regarding stem cell therapy in animal model of skin flap for the primary outcome of survival rate of flap. [file 13287_2020_2103_MOESM6_ESM.png]

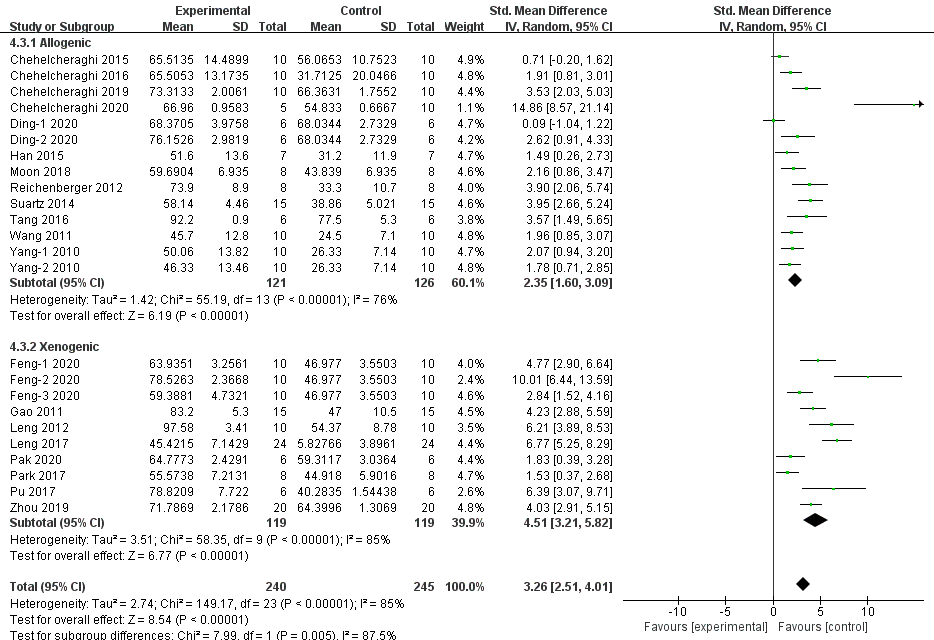

Supplement: Supplementary file 7 — Additional file 7: Figure S6. Subgroup analyses of transplant types regarding stem cell therapy in animal model of skin flap for the primary outcome of survival rate of flap. [file 13287_2020_2103_MOESM7_ESM.png]

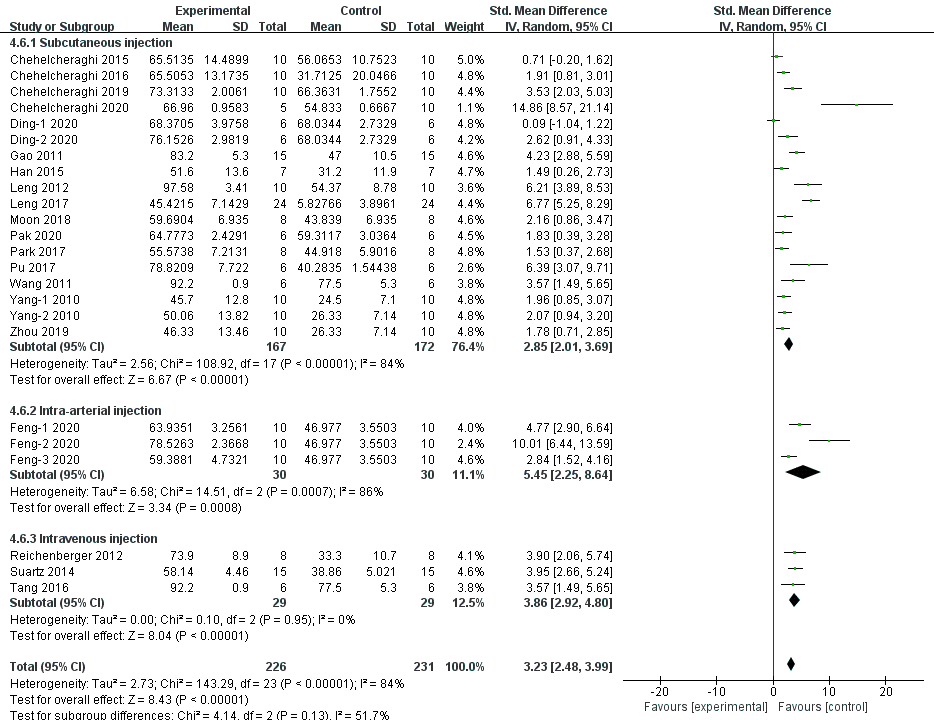

Supplement: Supplementary file 8 — Additional file 8: Figure S7. Subgroup analyses of method of administration of stem cells regarding stem cell therapy in animal model of skin flap for the primary outcome of survival rate of flap. [file 13287_2020_2103_MOESM8_ESM.png]
